# Navigation activities in an organized colorectal cancer screening program improve follow-up colonoscopy completion

**DOI:** 10.1038/s41598-026-44477-6

**Published:** 2026-03-14

**Authors:** Amanda Kimura, Amy Peck, Ari Bell-Brown, Kaitlin Todd, Nkem O. Akinsoto, Victoria Fang, Jerry Wood, Rachel B. Issaka

**Affiliations:** 1 Hutchinson Institute for Cancer Outcomes Research, Fred Hutchinson Cancer Center, Seattle, WA USA; 2 UW Medicine Primary Care and Population Health, University of Washington Medicine, Seattle, WA USA; 3 Division of General Internal Medicine, University of Washington School of Medicine, Seattle, WA USA; 4 Public Health Sciences & Clinical Research Divisions, Fred Hutchinson Cancer Center, Seattle, WA USA; 5 Division of Gastroenterology, University of Washington School of Medicine, Seattle, WA USA; 6 1100 Fairview Ave. N., M/S: M3-B232, Seattle, WA 98109 USA

**Keywords:** Colorectal cancer, Screening, Fecal immunochemical test, Colonoscopy, Patient navigation, Abnormal test follow-up, Cancer, Gastroenterology, Oncology

## Abstract

Patient navigation is a promising intervention that could improve follow-up of abnormal fecal immunochemical test (FIT) results in colorectal cancer (CRC) screening. We describe changes in navigation activities in an organized screening program aimed to improve follow-up colonoscopy completion. Between 2022 and 2023, we decreased the time between an abnormal FIT result and contact from the program’s patient navigator from 3 months to 1 month and provided the patient navigator with direct access to schedule colonoscopies at two endoscopy sites. We conducted a pre-post analysis that examined the proportion of patients with abnormal FIT results who completed a colonoscopy, were referred for a colonoscopy, time to referral, time to colonoscopy, and colonoscopy outcomes including CRC diagnoses. Our analysis included 368 patients with abnormal FIT results: 175 in 2022 and 193 in 2023. After changes to navigation activities, colonoscopy completion within 1 year increased by 22.9% points (42.9% to 65.8%; *p* < 0.001). In 2022, the median time (interquartile range; IQR) to colonoscopy was 103.5 (IQR 60.2-161.5) days. In 2023, the median time to colonoscopy was 99.0 (IQR 52.0-150.0) days. Differences in the proportion of patients referred to colonoscopy, time to referral, and time to colonoscopy were not statistically significant. Patient navigation beginning within 1 month of an abnormal FIT result and granting a patient navigator direct access to the endoscopy scheduling template increased 1-year colonoscopy completion. Understanding navigation activities in CRC screening programs could inform broader adoption of practices that are associated with increased follow-up colonoscopy completion.

**Supplementary Information:**

The online version contains supplementary material available at 10.1038/s41598-026-44477-6.

## Introduction

Colorectal cancer (CRC) is the third most common cancer and the second leading cause of cancer deaths in the United States (U.S.)^1^. Screening effectively detects CRC early, however, only 59% of adults ages 45 to 75 years old are up-to-date with screening which is below the National Colorectal Cancer Roundtable goal of 80%^2–4^. While colonoscopy remains the primary modality for CRC screening in the U.S., non-invasive stool-based testing, such as fecal immunochemical tests (FIT) are frequently used^5,6^. Implementing FIT through organized screening programs additionally increases screening participation in health systems.

For FIT to be successful, a follow-up colonoscopy must be completed after an abnormal result. Missed colonoscopies after an abnormal FIT result doubles the risk of dying from CRC^7^. Professional societies have recommended an 80% colonoscopy completion rate goal for patients with abnormal FIT results^8^. However, inadequate follow-up colonoscopies have been reported across several health systems^9,10^. Patient-level barriers (e.g. fear of colonoscopy), provider-level barriers (e.g. lack of result awareness) and system-level barriers (e.g. scheduling challenges) have all been associated with incomplete follow-up^11,12^.

Navigation has emerged as a promising intervention to improve follow-up of abnormal FIT results. Navigation is defined as the role and activities that enable people to overcome barriers and facilitate access to quality care across the cancer care continuum and has effectively improved participation in different cancer screening programs^13–15^. More recently, patient navigation has been shown to increase follow-up colonoscopy after abnormal FIT results either as a stand-alone intervention or when paired with other interventions^16,17^.

Despite the promise of navigation, implementation details are under-reported^13^. Understanding the elements needed in a navigation program to successfully increase follow-up colonoscopy completion may help address the broader challenge of inadequate follow-up of non-invasive screening strategies. This quality improvement initiative aimed to enhance follow-up colonoscopy completion after abnormal FIT results by modifying navigation timing and granting navigators scheduling access. Consistent with SQUIRE 2.0 principles, in this paper we describe the local context, intervention components, outcomes, and process measures used to assess changes over time.

## Methods

### CRC program setting and patient population

This quality improvement initiative occurred in the Fred Hutchinson Cancer Center / UW Medicine CRC screening program. UW Medicine is the health system affiliated with the University of Washington (UW) and includes 5 medium-to-large clinic networks. Across ambulatory practices, UW Medicine serves about 330,000 patients in over 30 primary care practices. Details about the CRC screening program have been previously described^18^. Patients who received outreach through the mailed FIT campaign and had an abnormal FIT result between March 2022 and December 2023 were included in this analysis. The study was approved by the Fred Hutchinson Cancer Center Institutional Review Board. The Fred Hutchinson Cancer Center Institutional Review Board also granted a waiver of informed consent, as the study was minimal risk, was incorporated into existing clinical operations, and would have been impractical to carry out without the waiver. All methods followed this review board’s guidelines and regulations for human subjects research.

### Data sources

UW Medicine is supported by an integrated electronic health record (EHR), Epic, which is linked to other data sources including laboratory data and colonoscopies. We identified the eligible patient population from EHRs and obtained colonoscopy data through July 2024. We supplemented EHR with data from the CRC screening program database, which includes all patients who receive outreach through the screening program.

### Referral process for abnormal FIT

Primary care physicians (PCPs) are responsible for notifying patients of abnormal FIT results, the need for follow-up testing, and placing colonoscopy referrals to the gastroenterology (GI) team. Colonoscopy referrals are reviewed by GI providers for appropriateness and to assess sedation needs. Patients are then scheduled to complete the procedure at one of five endoscopy sites within the healthcare system based on preference and availability. These steps are archived and searchable using EHR tools. Notably, UW Medicine is an open healthcare system, and some patients opt to complete procedures elsewhere. In these instances, results from the outside healthcare system are scanned in the EHR or extracted from Care Everywhere - a feature within the Epic EHR that enables healthcare providers to securely exchange patient information across healthcare organizations.

### Navigation

Navigation was defined as guiding a patient from abnormal FIT to colonoscopy completion by helping identify barriers to follow-up and connecting patients with services as needed. In our health system, navigation is completed by a lay health worker, embedded within the CRC screening program, with a background in information sciences and prior experience as a patient care coordinator. The navigator in our program is a full-time employee and a member of the University of Washington Population Health and Primary Care team. The navigator received prior training on best practices for outreach to patients, CRC screening options, and EHR documentation. They contacted patients by telephone or EHR patient portal messaging.

The navigator used a weekly tracking spreadsheet to identify patients with abnormal FIT results for outreach. Activities included (1) confirming that referrals for colonoscopy were appropriately placed, (2) reviewing the EHR to determine colonoscopy completion status, (3) contacting patients to assess and resolve barriers to colonoscopy completion, (4) connecting patients to GI clinic schedulers as needed, and (5) connecting patients to non-GI resources as needed (e.g. financial aid and transportation) (Supplemental Fig. 1).

In 2022, based on a consensus workflow for follow-up of abnormal FIT results developed by the CRC screening program in partnership with primary care teams, navigation began 3 months after a patient received an abnormal FIT result. The 3-month window before navigation was due to primary care clinics desire to have time to contact their patients prior to outreach. During this time, the navigator did not have direct access to the colonoscopy schedule, but they connected patients to GI schedulers who could assist with this step. The navigator tracked follow-up colonoscopy completion monthly through the EHR for 12 months or until a follow-up colonoscopy was completed or a reason documented explaining why follow-up was not indicated (e.g., decline in health status). Navigation ended when a patient completed a colonoscopy, declined the colonoscopy, had a documented reason for lack of follow-up, or after 12 navigation attempts (1 calendar year).

In 2023 we updated the abnormal FIT workflow in partnership with primary care to enable navigation outreach to begin 1 month (vs. 3 months) after an abnormal FIT result. Navigators also received training and were granted access to scheduling colonoscopies at two endoscopy sites within the healthcare system. At the remaining endoscopy sites, the navigator continued to work with schedulers to flag patients with abnormal FIT results who needed assistance scheduling colonoscopies. Other components of the 2023 navigation workflow remained the same.

### Outcomes & co-variates

Our primary outcome was the proportion of individuals who completed a follow-up colonoscopy within 1 year of an abnormal FIT result. Secondary outcomes included proportion of individuals referred for a colonoscopy within 1 year of an abnormal FIT result, time to colonoscopy referral from the date of the abnormal FIT result, and time to first colonoscopy completion from the date of the abnormal FIT result. If a referral was received before the abnormal FIT result, then both the referral and any subsequent colonoscopy were excluded, as they were not considered to be due to the program’s intervention; similarly, colonoscopies completed before the abnormal FIT were also excluded.

We also examined colonoscopy outcomes including CRC, advanced precancerous lesions (tubular adenomas $\ge$1 cm, tubulovillous adenomas and high-grade dysplasias), non-advanced precancerous lesions (tubular adenomas <1 cm, sessile serrated adenomas), or negative for colorectal neoplasia (normal)^19^.

### Statistical analysis

Primary and secondary outcomes and patient demographic variables were described using proportions, medians, and interquartile ranges (IQR) and tested using Wilcoxon rank sum test, Chi squared analysis, and Fisher’s exact test as appropriate (Table 1). The between-year difference in proportions was determined, and the inverse of between-year difference was used to estimate the number needed to navigate. Multivariate logistic regression, including year as a covariate, modelled screening colonoscopy completion within 1 year of an abnormal FIT result as the outcome of interest. Accompanying adjusted odds ratios (aOR), 95% confidence interval (CI), and p values were reported in all instances and p values < 0.05 were considered statistically significant. Following Centers for Medicare and Medicaid Services (CMS) best practices, figures and tables have been censored for small cell sizes under 11. Analyses were performed using R version 4.4.2.

**Table 1:** Demographics of patients with abnormal fecal immunochemical test (FIT) results.

Category	Sub-group	2022	2023	p
N Patients with abnormal FIT: n (%)		175	193	
Age	Median (IQR)	59.0 (54.0 to 67.0)	55.0 (49.0 to 65.0)	< 0.001
Age by group	45–49	N/A	55 (28.5)	< 0.001
	50–64	116 (66.3)	88 (45.6)	
	65+	59 (33.7)	50 (25.9)	
Sex	Male	105 (60.0)	109 (56.5)	0.563
	Female	70 (40.0)	84 (43.5)	
Ethnicity	Non-Hispanic	153 (87.4)	163 (84.5)	0.614
	Hispanic	*	16 (8.3)	
	Unknown	12 (6.9)	14 (7.3)	
Race	White	121 (69.1)	131 (67.9)	0.586
	Asian	15 (8.6)	24 (12.4)	
	Black	18 (10.3)	20 (10.4)	
	Other/Unknown	21 (12.0)	18 (9.3)	
Primary language	English	172 (98.3)	169 (87.6)	< 0.001
	Other	*	24 (12.4)	
Insurance type	Commercial	66 (37.7)	102 (52.8)	< 0.001
	Medicaid	29 (16.6)	31 (16.1)	
	Medicare	76 (43.4)	46 (23.8)	
	Other/Unknown	*	14 (7.3)	
Marital status	Partnered	83 (47.4)	85 (44.0)	0.037
	Without Partner	81 (46.3)	80 (41.5)	
	Other/Unknown	11 (6.3)	28 (14.5)	
Last primary care visit	<=12 mo	139 (79.4)	151 (78.2)	0.234
	13–36 mo	32 (18.3)	31 (16.1)	
	No Encounter w/in 36 mo	*	11 (5.7)	
Colonoscopy referral within 12 months	No	40 (22.9)	30 (15.5)	0.099
	Yes	135 (77.1)	163 (84.5)	
Time to referral	Median (IQR)	4.0 (1.0 to 20.0)	5.0 (2.8 to 10.0)	0.679
Colonoscopy receipt within 12 months	No	100 (57.1)	66 (34.2)	< 0.001
	Yes	75 (42.9)	127 (65.8)	
Time to colonoscopy	Median (IQR)	103.5 (60.2 to 161.5)	99.0 (52.0 to 150.0)	0.284

* Per CMS rules, cells with *N* < 11 must be censored, collapsed into another category, or coarsened appropriately for public sharing. Some group categories as N/A as no patients met the criteria.

Demographic groups were tested for differences between 2022 and 2023 for proportion of group size (group distribution) using Fisher’s exact test for categorical variables with small cell size issues (*n* < 5), two-sample test for equality of proportions (continuity corrected) (also called Chi squared analysis) for categorical variables without small cell size issues, and Wilcoxon Rank Sum Test (also called Mann-Whitney U) test for continuous variables (due to lack of normality).

Patient demographic variables included age, sex, race, ethnicity, primary insurance provider (commercial, Medicaid, Medicare or other/unknown), primary language, marital status (Partnered- domestic partner, married, significant other; Without partner – divorced, widowed, separated, single; Other).

## Results

### Patient population

In 2022, of the 2,874 FITs completed through mailed outreach, 175 (6.1%) patients had an abnormal result. The median age of patients with an abnormal result was 59.0 years (IQR 54.0–67.0). In 2023, of the 4,925 FITs completed through mailed outreach, 193 (4.0%) patients had an abnormal result. The median age was 55.0 years (49.0–65.0). In both years, most patients were male, non-Hispanic, White, and identified English as their primary language (Table 1).

### 2022 abnormal FIT follow-up

In 2022, 42.9% (*n* = 75/175) of patients completed a colonoscopy following an abnormal FIT result within 1 year. The proportion of patients referred within 1 year after an abnormal result was 82.9% (*n* = 145/175). The median time from abnormal FIT to colonoscopy referral was 4.0 (IQR 1.0–20.0) days. The median time from abnormal FIT to colonoscopy completion was 103.5 (IQR 60.2-161.5) days.

### 2023 abnormal FIT follow-up

In 2023, 65.8% (*n* = 127/193) of patients completed a colonoscopy within 1 year following an abnormal FIT. The proportion of patients referred within 1 year after an abnormal result was 89.1% (*n* = 172/193). The median time from abnormal FIT to colonoscopy referral was 5.0 (IQR 2.8–10.0) days. The median time from abnormal FIT to colonoscopy completion was 99.0 (IQR 52.0-150.0) days.

### Comparison between 2022 and 2023

**Fig. 1:**
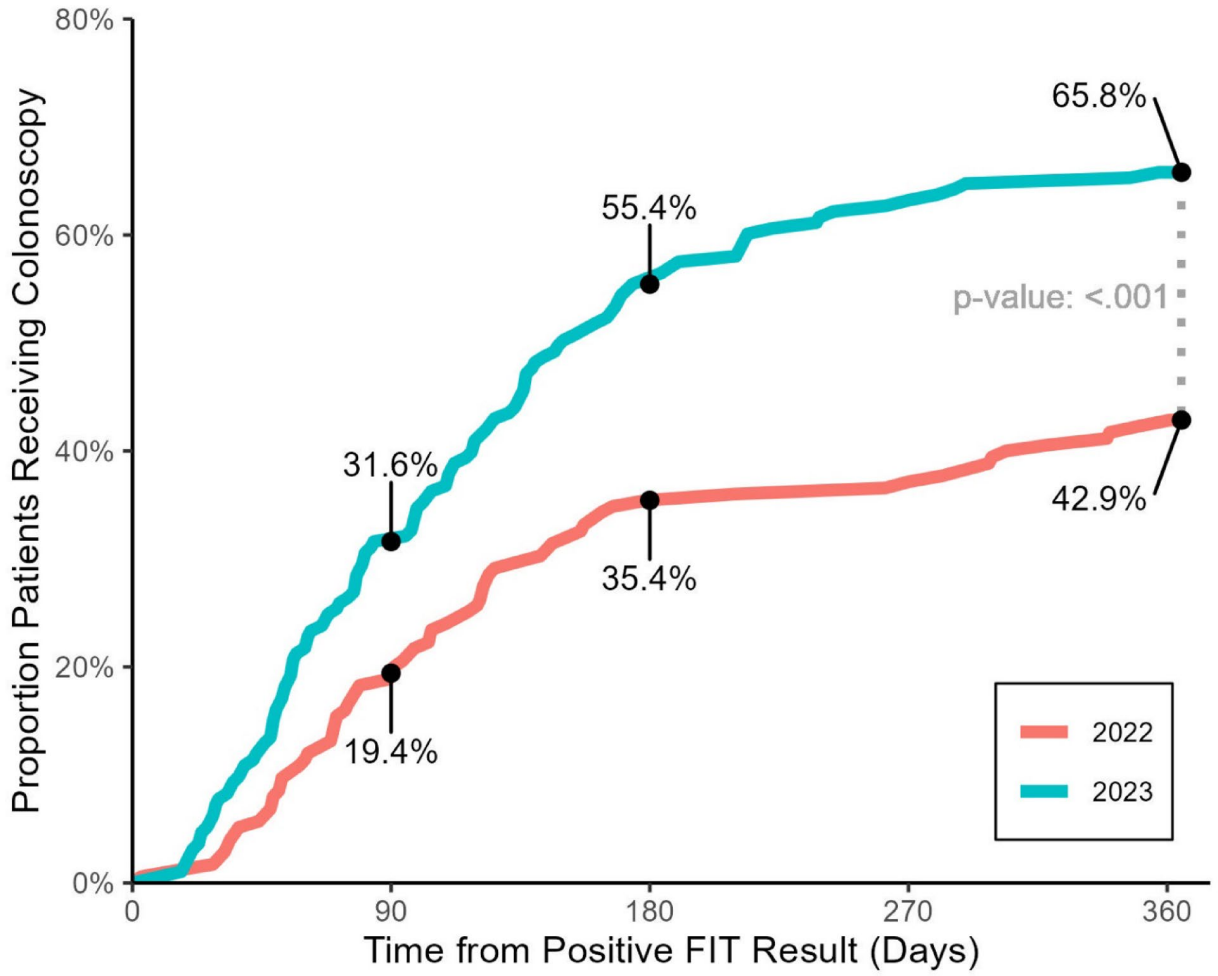
Time-to-event graph of patients receiving colonoscopy after an abnormal fecal immunochemical test (FIT). Cumulative portion of program patients with abnormal FIT results receiving colonoscopy. At 1 year, 42.9% of patients in the 2022 program had received this follow-up which increased to 65.8% in the 2023 program; a two-sample test for equality of proportions (continuity corrected) found this constituted a significant difference in proportion receiving colonoscopy follow-up at 1 year between program years (p = < 0.001).

**Fig. 2:**
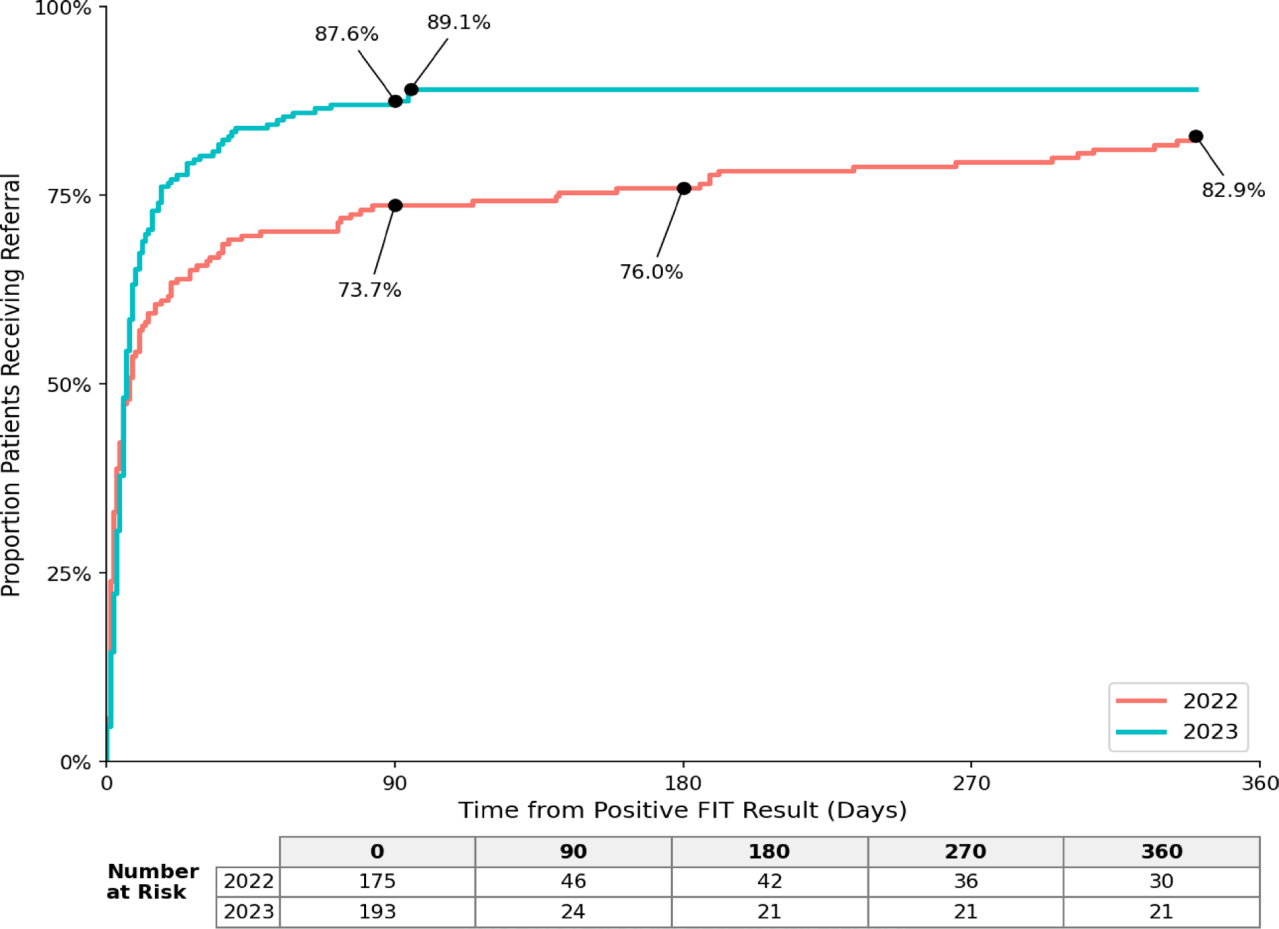
Time-to-event graph of patients receiving referral after an abnormal fecal immunochemical test (FIT). Cumulative portion of program patients with abnormal FIT results receiving colonoscopy referrals. At 1 year, 82.9% of patients in the 2022 program had received referrals which increased to 89.1% in the 2023 program; a two-sample test for equality of proportions (continuity corrected) found this was not a significant difference in proportion receiving colonoscopy referrals at 1 year between program years (*p* = 0.09).

After implementing changes to navigation, follow-up colonoscopy completion within 1 year increased by 22.9% points (*p* < 0.001) (Fig. 1). Colonoscopy referral within 1 year increased by 6.2% points (*p* = 0.09). Time to referral increased by 1 day (*p* = 0.68), but notably 67.4% (*n* = 130/193) of patients received referrals within 10 days in 2023 versus 65.7% (*n* = 115/175) within 30 days in 2022. The proportion of patients receiving a referral within 30 days and 90 days of abnormal result was higher in 2023 than in 2022 and time to colonoscopy completion decreased by 4.5 days (*p* = 0.29) (Fig. 2).

Multivariate analysis found patients with an abnormal FIT result in 2023, were more likely to complete a follow-up colonoscopy while patients with other/unknown insurance or lack of partner were less likely to complete follow-up colonoscopy. There were no other statistically significant differences in colonoscopy completion across demographic groups (Supplemental Table 1).

The number needed to receive earlier navigation after an abnormal result to complete an additional colonoscopy was 4.4.

### Colorectal cancer screening outcomes

In 2022, among patients who completed a colonoscopy, 8.0% (*n* = 6/75) were diagnosed with CRC, 32.0% (*n* = 24/75) had advanced precancerous lesions, 38.7% (*n* = 29/75) had non-advanced precancerous lesions, and 20.0% (*n* = 15/75) had normal findings.

**Fig. 3:**
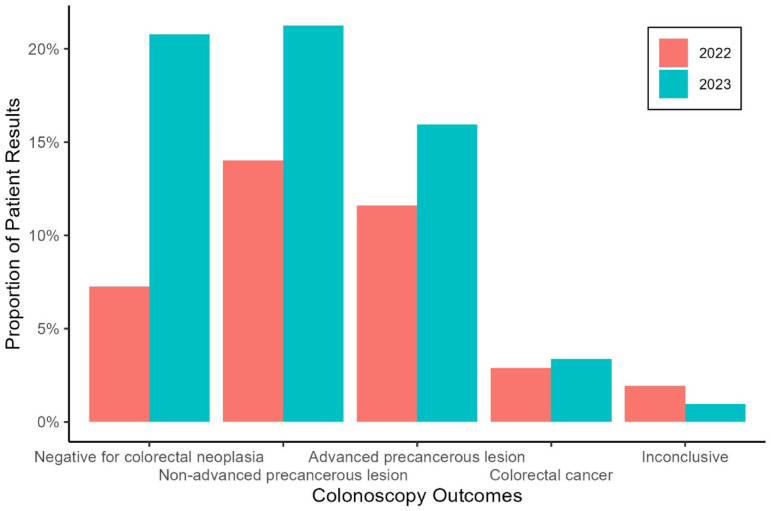
Colonoscopy outcomes of patients with an abnormal fecal immunochemical test (FIT) result. Portion of program patients with abnormal FIT results who subsequently received a follow up colonoscopy with pathology results.

In 2023, among patients who completed follow-up colonoscopies, 5.5% (*n* = 7/127) were diagnosed with CRC, 26.0% (*n* = 33/127) had advanced precancerous lesions, 34.6% (*n* = 44/127) had non-advanced precancerous lesions, and 33.9% (*n* = 43/127) were negative for colorectal neoplasia (Fig. 3).

## Discussion

In a pre-post analysis of an organized CRC screening program, starting patient navigation at 1 month, rather than 3 months, after an abnormal FIT result, and providing navigators direct access to colonoscopy scheduling, was associated with a 23-percentage point higher proportion of patients completing follow-up colonoscopy. However, differences in the proportion of patients referred for follow-up colonoscopy, the time to referral, and time to colonoscopy completion were modest and not statistically significantly different.

Patient navigation is a potentially promising intervention to address multiple barriers to follow-up colonoscopy, including patient, provider, and system-level barriers. Existing literature shows that patient navigation is effective in increasing uptake in cancer screening, decreasing time from screening to diagnosis, and improving treatment initiation^20,21^. Patient navigation has also shown promise for increasing follow-up colonoscopy completion after abnormal FIT results, but results vary by patient population and details of the navigation program are rarely reported. For example, in an integrated safety-net healthcare system, a quality improvement study using patient navigators increased follow-up colonoscopy completion by 5.4% points (40.6% to 46%)^16^. However, in a different setting, an integrated managed care health system, navigation improved follow-up colonoscopy completion by 11.0% points compared to the control group^22^.

To increase adoption of navigation programs, implementation and contextual details must be more broadly reported^23^. Similar to our study, Shareef, et al. detailed their Plan-Do-Study-Act approach in making changes to their patient navigation program. After standardizing their navigation protocols, the health system observed a 17.0% point increase in follow-up colonoscopy completion^24^. In another study, over a 10-year period, Kaiser Permanente Northern California tracked strategies implemented within a specific window and measured follow-up colonoscopy rates^25^. While these strategies were broad and included tracking abnormal FIT patients, early contact to schedule colonoscopies, and increasing colonoscopy capacity; they demonstrated that these combined interventions increased follow-up colonoscopy by 16.0% points over a 10-year period. Because implementing all these interventions might be challenging in resource-constrained settings, understanding the individual contributions of these interventions, including navigation, could help health systems prioritize use of limited resources. Such information is also critical to informing future Centers for Medicare & Medicaid Services (CMS) coverage decisions. CMS reimbursement for navigation services in cancer care has increased the feasibility for health systems to invest in navigator programs, and emerging evidence may support expansion of CMS coverage to include navigation services for cancer screening.

Increased use of direct access endoscopy or granting more members of the healthcare team access to endoscopy scheduling are other strategies that could potentially improve follow-up of abnormal non-invasive screening tests. This could help patients and health systems overcome scheduling challenges which include but are not limited to inaccurate patient contact information, difficulty reaching patients after multiple attempts, and patient cancellations without rescheduling^12^. In one large teaching hospital, a direct access colonoscopy scheduling system coupled with patient navigation for primarily Black and Hispanic patients, led to 66% of referred patients ultimately completing a colonoscopy^26^. Another study found that screening and surveillance colonoscopy completion rates were similar in patients referred through direct access endoscopy versus those who were scheduled during in-office visits^27^. While these studies were not restricted to patients with abnormal FIT results, models that decrease barriers to colonoscopy scheduling could be applied to CRC programs that prioritize non-invasive screening tests.

In the setting of colonoscopy capacity limitations, which were exacerbated by the COVID-19 pandemic, it is important to consider approaches to screening that maximize the use of available colonoscopy resources. Organized stool-based testing programs play an important role in ensuring continued access to screening while prioritizing colonoscopy for those with abnormal tests and higher risk for CRC. However, suboptimal colonoscopy completion rates after abnormal FIT results persist in multiple healthcare settings. For these reasons, implementing and evaluating interventions that increase colonoscopy completion are necessary to improve patient outcomes and to make the most efficient use of limited colonoscopy resources.

Our study has limitations. First, this was a single-center, pre-post analysis of a quality improvement initiative. While our findings are promising, they may not be generalizable to all clinical settings and larger studies are needed to validate our findings. Notably, our centralized program enabled comprehensive tracking of patients and documentation of follow-up colonoscopy completion status. Our data suggests that the number needed to navigate to obtain an additional completed colonoscopy is 4, which is reasonable on face value. Second, while we purposefully limited our analysis to a 2-year period to limit the influence of other secular trends that might influence colonoscopy completion (e.g. waning effect of the COVID-19 pandemic), it is possible that our study did not completely capture all influencing factors. We do believe that this analysis provides insights into the effect that programmatic changes made on follow-up colonoscopy completion in our health system. Third, we did not evaluate navigation burden or resource diversion. Given this persistent challenge in many health systems, we hope to examine these effects in future analysis. Finally, despite improvements, these interventions did not reach the 80% follow-up colonoscopy goal. Notably, 87.6% of patients were referred for a follow-up colonoscopy in 2023 within 90 days of this abnormal result. However, it is likely that additional interventions are needed to ensure that all referred patients ultimately complete a follow-up colonoscopy. Our findings are encouraging and will be refined towards achieving the 80% follow-up benchmark as our navigation program continues to evolve.

In summary, in an organized CRC screening program, shortening the interval between an abnormal FIT result and navigation outreach and granting navigators access to the endoscopy scheduling platform was associated with a significantly higher proportion of patients completing follow-up colonoscopy. By sharing the details of navigation activities used in this CRC screening program, we aim to foster increased adoption in other settings to potentially improve follow-up colonoscopy completion; a critical step in ensuring all patients realize the full benefits of non-invasive CRC screening.

## Supplementary Information

Below is the link to the electronic supplementary material.

Supplementary Material 1

## Data Availability

The datasets generated and/or analysed during the current study are not publicly available due restrictions on sharing protected patient health information but are available from the corresponding author on reasonable request.

## Abbreviations

CRC: Colorectal cancer
FIT: Fecal immunochemical test
PCP: Primary care provider
EHR: Electronic health record
IQR: Interquartile range
