# Supplementary material for: Navigation activities in an organized colorectal cancer screening program improve follow-up colonoscopy completion
Source: Sci Rep. 2026 Mar 14;16:13509. doi: 10.1038/s41598-026-44477-6 (PMC13111698; doi:10.1038/s41598-026-44477-6)

Navigation Activities in an Organized Colorectal Cancer Screening Program Improve Follow-Up Colonoscopy Completion

**Supplementary Material**

**Supplemental Table 1**: Factors associated with completing a colonoscopy within one year of an abnormal fecal immunochemical test (FIT) result during the 2022 and 2023 programs

**Supplemental Figure 1**: Abnormal fecal immunochemical test (FIT) follow-up process map

**Supplemental Table 1.** Factors associated with completing a colonoscopy within one year of an abnormal fecal immunochemical test (FIT) result during the 2022 and 2023 programs

|  |  | **2022 & 2023** | | |
| --- | --- | --- | --- | --- |
|  |  | **Total Eligible: 368** | | |
|  |  | **N Colonoscopies: 202** | | |
| **Variable** | **Category** | **aOR** | **95% CI** | **p** |
| **Year** | 2022 | ref | - | - |
|  | 2023 | 2.91 | 1.8 - 4.78 | <0.001 |
| **Age** |  | 0.99 | 0.96 - 1.02 | 0.518 |
| **Sex** | Male | ref | - | - |
|  | Female | 0.65 | 0.41 - 1.04 | 0.072 |
| **Ethnicity** | Non-Hispanic | ref | - | - |
|  | Hispanic | 1.24 | 0.42 - 3.83 | 0.700 |
|  | Unknown | 0.46 | 0.16 - 1.32 | 0.153 |
| **Race** | White | ref | - | - |
|  | Asian | 1.38 | 0.64 - 3.11 | 0.421 |
|  | Black | 0.61 | 0.28 - 1.3 | 0.205 |
|  | Other/Unknown | 1.33 | 0.53 - 3.35 | 0.545 |
| **Primary Language** | English | ref | - | - |
|  | Other | 0.53 | 0.18 - 1.48 | 0.225 |
| **Insurance Type** | Commercial | ref | - | - |
|  | Medicaid | 0.66 | 0.33 - 1.29 | 0.223 |
|  | Medicare | 0.66 | 0.34 - 1.27 | 0.216 |
|  | Other/Unknown | 0.29 | 0.09 - 0.83 | 0.023 |
| **Marital Status** | Partnered | ref | - | - |
|  | Without Partner | 0.51 | 0.3 - 0.86 | 0.012 |
|  | Other/Unknown | 0.56 | 0.25 - 1.27 | 0.159 |
| **Last Primary Care Visit** | <=12 mo | ref | - | - |
|  | 13-36 mo | 1.29 | 0.69 - 2.44 | 0.428 |
|  | No Encounter w/in 36 mo | 1.58 | 0.48 - 5.72 | 0.465 |

**Supplemental Figure 1**. Abnormal fecal immunochemical test (FIT) follow-up process map


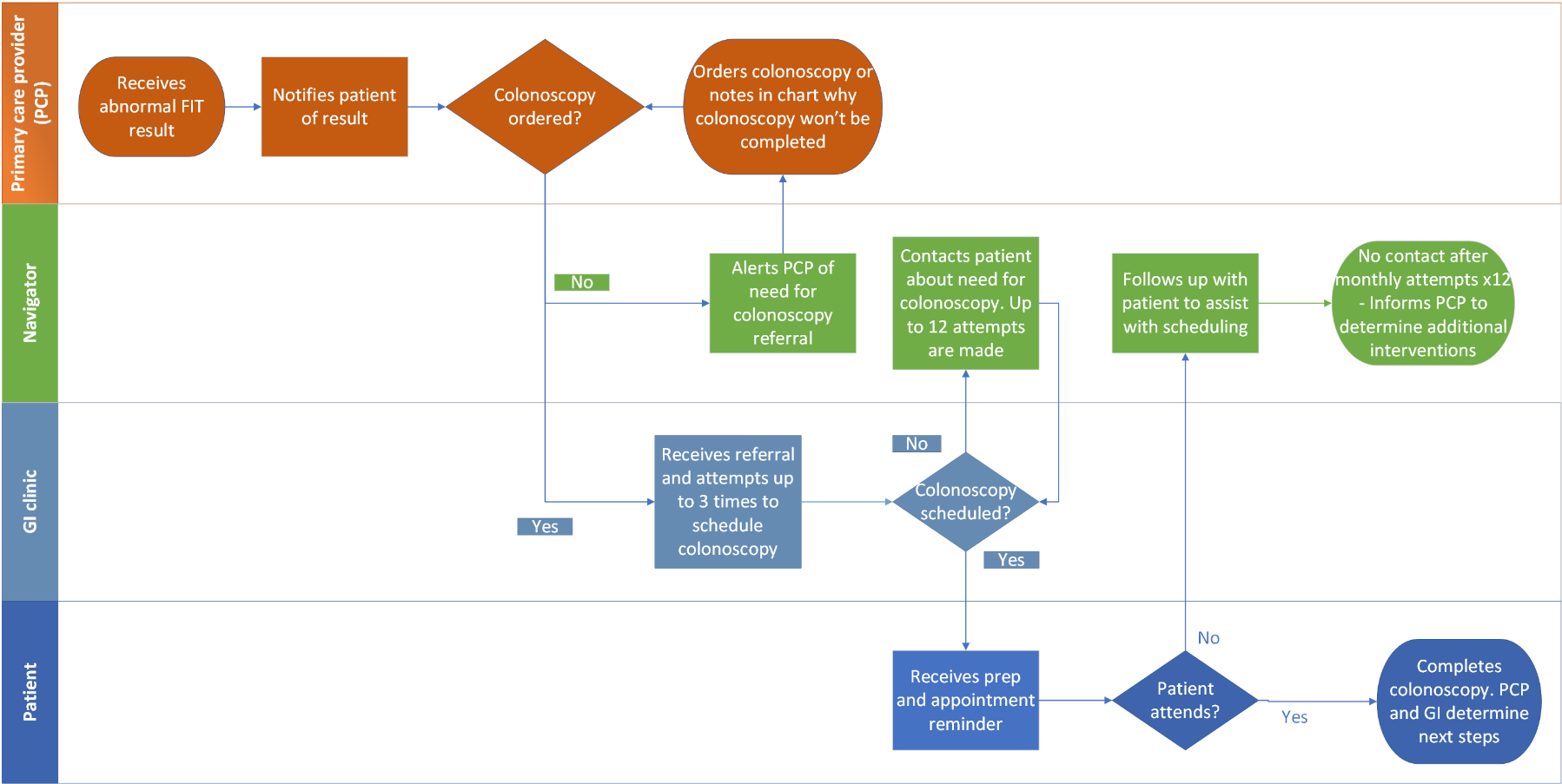

Supplement: Supplementary file 1 — Supplementary Material 1 [file 41598_2026_44477_MOESM1_ESM.docx]
